# Oxidative Transformation of Dihydroflavonols and Flavan-3-ols by Anthocyanidin Synthase from *Vitis vinifera*

**DOI:** 10.3390/molecules27031047

**Published:** 2022-02-03

**Authors:** Jia-Rong Zhang, Claudine Trossat-Magnin, Katell Bathany, Luc Negroni, Serge Delrot, Jean Chaudière

**Affiliations:** 1 Chimie et Biologie des Membranes et des Nano-Objets (CBMN, UMR 5248), Université de Bordeaux, 33615 Pessac, France; jiarong.zhang@gtiit.edu.cn (J.-R.Z.); k.bathany@cbmn.u-bordeaux.fr (K.B.); luc.negroni@igbmc.fr (L.N.); 2 Biotechnology and Food Engineering Program, Guangdong Technion-Israel Institute of Technology, Shantou 515063, China; 3 Institut des Sciences de la Vigne et du Vin (ISVV, UMR 1287), Université de Bordeaux, 33140 Villenave d’Ornon, France; claudine.trossat-magnin@u-bordeaux.fr (C.T.-M.); serge.delrot@u-bordeaux.fr (S.D.); 4 Institut de Génétique et de Biologie Moléculaire et Cellulaire (IGBMC, UMR7104), 1 Rue Laurent Fries, 67400 Illkirch-Graffenstaden, France

**Keywords:** anthocyanidin synthase, *Vitis vinifera*, 2-oxoglutarate, ascorbate, dihydroflavonol, flavanol, catechin, leucoanthocyanidin, structure-activity relationship, mass spectrometry

## Abstract

Twelve polyphenols from three distinct families (dihydroflavonols, flavan-3-ols, and flavanones) were studied as potential substrates of anthocyanidin synthase from *Vitis vinifera* (*Vv*ANS). Only flavan-3-ols of (2*R*,3*S*) configuration having either a catechol or gallol group on ring B are accepted as substrates. Only dihydroflavonols of (2*R*,3*R*) configuration are accepted as substrates, but a catechol or gallol group is not mandatory. Flavanones are not substrates of *Vv*ANS. HPLC and MS/MS analyses of the enzymatic products showed that the *Vv*ANS-catalyzed oxidative transformation of (+)-dihydroflavonols, such as dihydroquercetin, dihydrokaempferol and dihydromyricetin, leads only to the corresponding flavonols. Among the flavan-3-ols recognized as substrates, (+)-gallocatechin was only transformed into delphinidin by *Vv*ANS, whereas (+)-catechin was transformed into three products, including two major products that were an ascorbate–cyanidin adduct and a dimer of oxidized catechin, and a minor product that was cyanidin. Data from real-time MS monitoring of the enzymatic transformation of (+)-catechin suggest that its products are all derived from the initial C_3_-hydroxylation intermediate, i.e., a 3,3-gem-diol, and their most likely formation mechanism is discussed.

## 1. Introduction

Anthocyanins, an important subfamily of flavonoids, are among the most widely distributed water-soluble pigments in plants, and are largely responsible for the colors of flowers, fruits, and vegetables that range from orange or red to purple or blue. They have long been reported to fulfill multiple ecological and physiological functions within plants by contributing to their growth and subsistence in many ways. For example, they enable plants to maintain higher growth rates in metal-polluted soils and to postpone plant senescence under nitrogen deficiency or nutrient deficiency [1]. Moreover, they act as visual signals that attract pollinators and seed-dispersers, protect against pathogens and herbivores through phytoalexins and chemical repellents, act as photo-protecting screens against damage from intense light and UV-B radiation, and inhibit the overproduction of reactive oxygen species (ROS) by chelating metals and scavenging or destroying ROS [2,3,4,5,6,7,8,9]. When used as nutraceuticals, additional properties of interest include their specific interactions with target proteins [10]. The interest in anthocyanins has markedly increased with the recognition of their potential health benefits [11,12,13,14]. Numerous investigations of cell lines in vitro and animal models in vivo have been carried out during the past two decades, as well as epidemiological studies and clinical trials with human volunteers. Such studies have demonstrated that several of these natural dietary phytochemicals exhibit pharmacological properties that could be useful in anticancer [15,16], anti-inflammatory [17], or anti-obesity [18,19,20,21] strategies, as well as for cardioprotection [22] or to alleviate diabetes [23,24].

Despite this growing interest, some steps of the biosynthetic pathway of anthocyanins still need to be clarified. Beyond the biosynthesis of dihydroflavonols that involve chalcone synthase, chalcone isomerase, and flavanone-3-hydroxylase, it is generally recognized that at least three key enzymes are involved, including dihydroflavonol reductase (DFR), anthocyanidin synthase (ANS), and UDP-glucose: flavonoid 3-O-glucosyltransferase (UFGT) [25,26]. It is not doubtful that DFR activity is required upstream of ANS, which suggests that leucoanthocyanidins are physiological substrates of ANS. For example, treatment of *Vitis vinifera* cell suspension cultures with salicylic acid, a natural phytohormone that stimulates the production of anthocyanins and proanthocyanidins, was found to simultaneously induce the mRNA and protein accumulation of *Vv*CHS, *Vv*CHI, *Vv*DFR, and *Vv*ANS [27].

ANS is the first enzyme in the anthocyanin pathway, it yields anthocyanidins that can be further glycosylated by UFGT into anthocyanins. For years, it has been thought that the products of the in vivo DFR-catalyzed reduction of dihydroflavonols, namely leucoanthocyanidins, are the natural substrates of ANS, and that their oxidative transformation by ANS is the major physiological source of anthocyanidins in vivo. Solid evidence provided by previous functional genomic studies supported the specific requirement of the corresponding ANS gene in anthocyanidins biosynthesis [25,26,27,28,29,30,31], in agreement with the idea that ANS is indeed the enzyme that produces anthocyanidins, as its name suggests. However, the ANS-catalyzed oxidative transformation of leucoanthocyanidins has never been confirmed to be a major source of anthocyanidins in vivo. In a recent study, we showed that anthocyanidin synthase from *Vitis vinifera* (*Vv*ANS) could not produce cyanidin in vitro with the natural stereoisomer of leucocyanidin as the substrate [32], and it seems that this observation also applies to ANS from *Arabidopsis thaliana*, *Perilla frutescens*, and *Ginkgo biloba* [31,33,34,35,36,37], with which cyanidin was always observed as a minor product of the natural isomer of leucocyanidin in vitro. In addition, we showed with *Vv*ANS that only traces of cyanidin were produced in vitro from the unnatural stereoisomer, i.e., 2*R*,3*S*,4*S*-trans-leucocyanidin [32].

ANS is a member of the 2-oxoglutarate-dependent oxygenase superfamily, which includes other enzymes in the flavonoid biosynthetic pathway [38], and requires 2-oxoglutarate, iron(II), and ascorbate for its activity [36]. We found that *Vv*ANS is inactive in the absence of ascorbate and that its activity is almost optimal at pH 6.3 [39]. We showed that *Vv*ANS first produces a 3,3-gem-diol intermediate with each of the two stereoisomers of leucocyanidin [32], which suggested that C_3_-hydroxylation of its substrates is most likely its generic function.

In the present work, we show (i) that *Vv*ANS is active on dihydroflavonols that are only transformed into flavonols, and (ii) that the enzyme is more active on flavan-3-ols, from which anthocyanidins can be produced. Additionally, two byproducts are also observed upon enzymatic transformation of (+)-catechin, including an ascorbate–cyanidin adduct and a dimer of oxidized catechin.

## 2. Results

### 2.1. Transformation of Dihydroflavonols by VvANS

**Figure 1:**
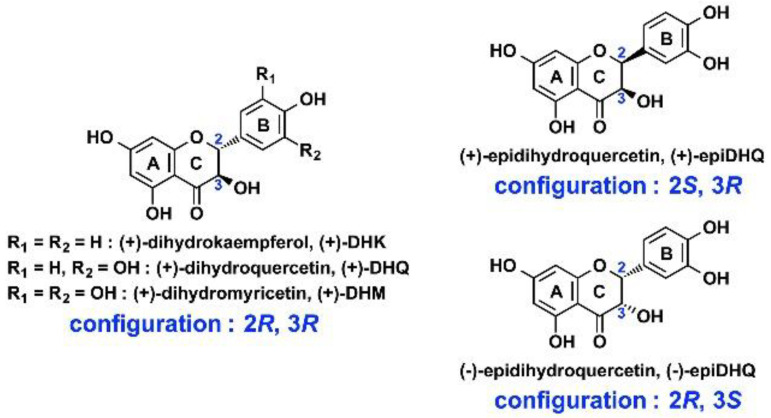
Structures of the five tested dihydroflavonols.

Dihydroflavonols constitute a subfamily of flavonoids; one of them, (+)-DHQ, which has a catechol group on ring B (Figure 1), is accepted as substrate by both AtANS [40] and *Vv*ANS [32]. Four other dihydroflavonols were tested, including (+)-DHK, (+)-DHM, (+)-epiDHQ, and (-)-epiDHQ.

#### 2.1.1. (+)-Dihydrokaempferol (DHK)

**Figure 2:**
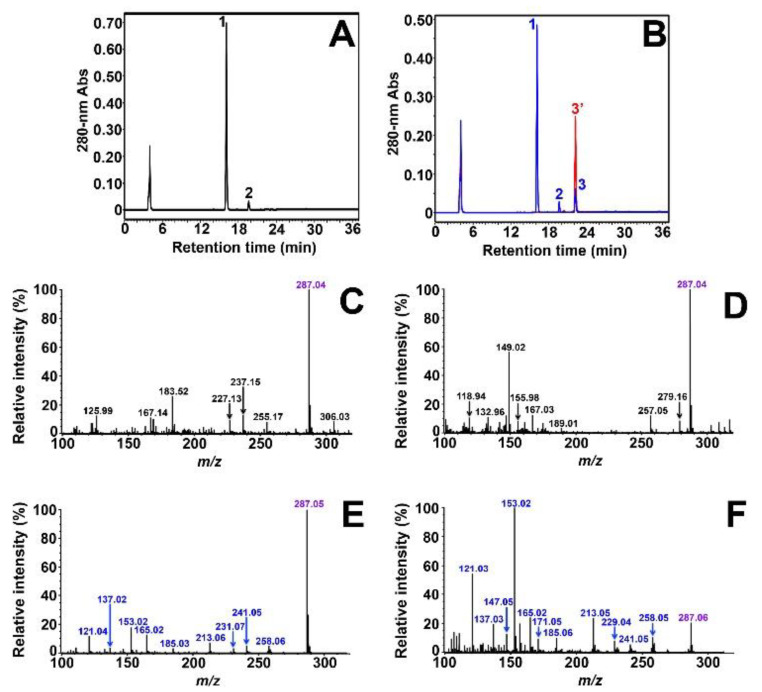
Characterization of *Vv*ANS product of (+)-DHK. **(A)** HPLC analysis of the commercial standard of 100 µM (+)-DHK in the reaction mixture without enzyme. **(B)** HPLC overlay chromatogram of the enzymatic degradation product of (+)-DHK (in blue) and the commercial standard of 100 µM kaempferol (in red). Peak 1 is residual (+)-DHK, peak 2 is the contaminant of commercial (+)-DHK, and peak 3 is the single enzymatic product of (+)-DHK. **(C)** MS analysis of collected peak 3. **(D)** MS spectrum of commercial kaempferol. **(E)** MS/MS fragmentation of the [M+H]^+^ ion with *m*/*z* 287.04 observed in (C). **(F)** MS/MS spectrum of commercial kaempferol. The two minor peaks observed at 4 min in (A) and (B) correspond to ascorbate present in the reaction mixture as a cofactor of *Vv*ANS.

(+)-DHK has a single phenolic hydroxyl on ring B (Figure 1), and its transformation by *Vv*ANS was studied first. As shown in Figure 2A, reverse-phase HPLC analysis of the commercial standard of (+)-DHK shows two peaks on the chromatogram. The major peak 1 (16.1 min) corresponds to (+)-DHK, and the small peak 2 (19.6 min) is a contaminant of the commercial product of (+)-DHK; this was unambiguously identified by reverse-phase HPLC and MS/MS as naringenin, using a commercial standard of (±)-naringenin (Figure S1). Upon incubation with *Vv*ANS (Figure 2B, in blue), a single product (peak 3, 22.3 min) was observed with the same retention time as that of a commercial standard of kaempferol (red peak 3′, 22.3 min). This product was collected during HPLC analysis, and its characterization by MS and MS/MS was subsequently carried out using commercial kaempferol as external standard. As shown in Figure 2C, a single major peak at *m*/*z* 287.04 is observed on the MS spectrum of peak 3, which should correspond to the [M+H]^+^ ion of the product. Therefore, the product visualized as peak 3 has a molecular weight of 286.03 Da, which is the same as that of kaempferol ([M+H]^+^, *m*/*z* 287.04) (Figure 2D). Further MS/MS fragmentation of the ion with *m*/*z* 287.04 yields several fragment ions (Figure 2E), which are very similar to those of the [M+H]^+^ ion of kaempferol (Figure 2F). We conclude that (+)-DHK is accepted by *Vv*ANS as a substrate to be exclusively transformed into kaempferol.

#### 2.1.2. (+)-Dihydromyricetin (DHM)

**Figure 3:**
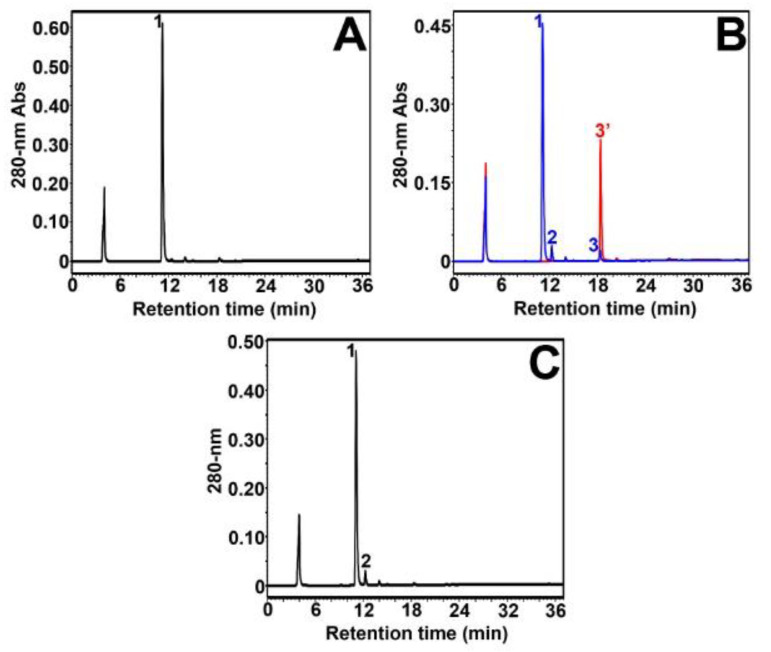
Reverse-phase HPLC analysis of *Vv*ANS product of (+)-DHM. (**A**) HPLC analysis of the commercial standard of 100 µM (+)-DHK in the reaction mixture containing no enzyme. (**B**) HPLC overlay chromatogram of the enzymatic degradation product of (+)-DHM (in blue) and the commercial standard of 100 µM myricetin (in red). (**C**) HPLC analysis of the non-enzymatic degradation of 100 µM (+)-DHM in the reaction mixture containing iron(II) salt but no enzyme. The minor peak observed at about 4 min corresponds to ascorbate.

The enzymatic transformation of (+)-DHM, a dihydroflavonol containing a gallol group on ring B (Figure 1), was studied next. As shown in Figure 3A, reverse-phase HPLC analysis of the commercial standard of (+)-DHM showed only a major peak (peak 1, 11.2 min) and no contaminant was observed. Upon incubation with *Vv*ANS, two small new peaks were observed (peaks 2 and 3, Figure 3B). The blue peak 3 observed at 18.4 min corresponded to the single enzymatic product of (+)-DHM, and it was assigned to myricetin based on the HPLC retention time of the corresponding commercial standard (red peak 3′, 18.4 min). Further MS and MS/MS analyses of the collected peak 3 revealed that its MS and MS/MS spectra were very similar to those of commercial myricetin (Figure S2). The blue peak 2 observed at 12.3 min was considered as a non-enzymatic product of (+)-DHM or a complex of (+)-DHM with iron(II), because this peak was again observed upon incubation of (+)-DHM in the reaction mixture containing iron(II) salt but no enzyme (Figure 3C). We conclude that (+)-DHM is a substrate of *Vv*ANS that gives myricetin as a single enzymatic product.

#### 2.1.3. Dihydroquercetin Stereoisomers (DHQ)

Two stereoisomers of (+)-DHQ, (+)-epiDHQ and (−)-epiDHQ, were then also tested as substrates of *Vv*ANS, and no enzymatic product was observed in either case (data not shown).

### 2.2. Transformation of Flavan-3-ols by VvANS

**Figure 4:**
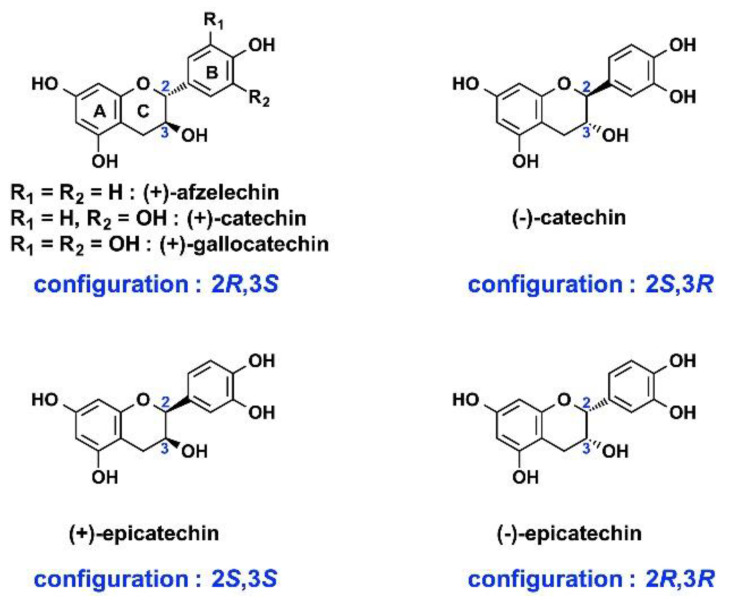
Structures of the six tested flavan-3-ols.

Flavan-3-ols, also known as flavanols, are characterized by the presence of an aliphatic hydroxyl group at C_3_ and an unsubstituted C_4_-methylene. It has been reported that one of them, (+)-catechin, which bears two phenolic hydroxyl groups (catechol) on ring B, is a substrate of ANS from Gerbera hybrida [41]. (+)-Catechin was therefore tested as substrate of *Vv*ANS; further, five other flavan-3-ols were also tested, including (+)-afzelechin (−), -catechin, (+)-epicatechin, (-)-epicatechin, and (+)-gallocatechin (Figure 4).

#### 2.2.1. (+)-Catechin

**Figure 5:**
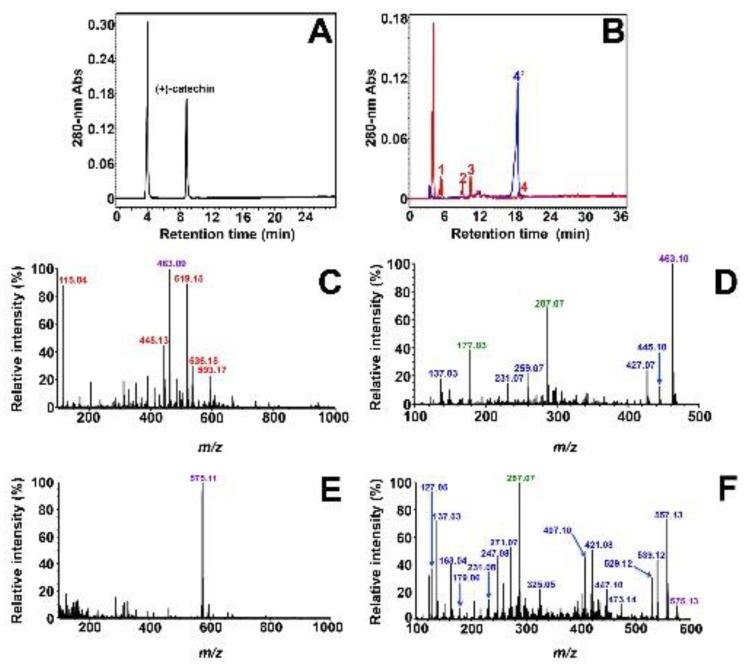
Characterization of *Vv*ANS product of (+)-catechin. (**A**) Reverse-phase HPLC analysis of the non-enzymatic degradation of 100 µM (+)-catechin in the reaction mixture without enzyme. (+)-Catechin is eluted at 9.0 min, and no product is observed upon incubation in the absence of *Vv*ANS. (**B**) HPLC overlay chromatogram of the enzymatic degradation products of (+)-catechin (in red) and the commercial standard of 100 µM cyanidin chloride (in blue). (**C**) MS analysis of collected peak 1. (**D**) MS/MS fragmentation of the [M+H]^+^ ion with *m*/*z* 463.09 observed in (C). (**E**) MS analysis of collected peak 3. (**F**) MS/MS fragmentation of the [M+H]^+^ ion with *m*/*z* 575.11 observed in (**E**). The two large peaks observed at 4.0 min in (**A**,**B**) correspond to ascorbate.

The transformation of flavan-3-ols by *Vv*ANS was first carried out using (+)-catechin as substrate, and a control experiment was performed in the absence of *Vv*ANS (Figure 5A). As shown in Figure 5B (in red), four peaks (peaks 1–4) are observed upon incubation with *Vv*ANS, among which peak 2 (9 min) corresponds to residual (+)-catechin, whereas the other three peaks correspond to new enzymatic products. As shown in Figure 5B (in red), the small peak 4 (18.6 min) could be assigned to cyanidin based on the HPLC retention time of the commercial standard of cyanidin chloride (blue peak 4′, 18.5 min), and further MS analyses of the collected peak 4 confirmed that its MS and MS/MS spectra were very similar to those of commercial cyanidin chloride (Figure S3).

The other two major products eluted at 5.5 and 10.5 min (peaks 1 and 3) were also collected and analyzed by MS and MS/MS. As shown in Figure 5C, six major ions, with *m*/*z* 115.04, 445.12, 463.09, 519.15, 536.17, and 593.17, were observed on the MS spectrum of collected peak 1, among which only the peak at *m*/*z* 463.09 corresponds to the [M+H]^+^ ion of the product; the five others are common contaminant ions encountered in positive ionization mass spectrometry [42]. Further MS/MS analysis of this [M+H]^+^ ion with *m*/*z* 463.09 yielded several fragment ions (Figure 5D), of which the two major ions with *m*/*z* 177.02 and 287.07 correspond respectively to the [M+H]^+^ ion derived from ascorbic acid (theoretical exact mass 176.03 Da) and M^+^ ion from cyanidin (theoretical exact mass 287.06 Da). We conclude that *m*/*z* 463.09 corresponds to the [M+H]^+^ ion of a covalent adduct of ascorbate with cyanidin (176.03 + 287.06 = 463.09 Da), which means that the product visualized as peak 1 should be an ascorbate–cyanidin adduct with a molecular weight of 462.08 Da.

As shown in Figure 5E, only the major ion with *m*/*z* 575.11 is observed on the MS spectrum of collected peak 3, which should be the [M+H]^+^ ion of the product. Further MS/MS analysis of this [M+H]^+^ ion with *m*/*z* 575.11 yielded several fragment ions (Figure 5F); among them, the most important fragment ion is that with *m*/*z* 287.07, which corresponds to the flavylium cation of cyanidin or an equivalent tautomeric form of the cation. Therefore, we conclude that *m*/*z* 575.11 most likely corresponds to the [M+H]^+^ ion of a dimer of cyanidin or oxidized catechin (theoretical exact mass 287.06 + 287.06 = 574.12 Da), which means that the product visualized as peak 3 should be a dimer of cyanidin or oxidized catechin with a molecular weight of 573.10 Da. Given the similarity of our results and those of Wellman et al. [41] with ANS from *Gerbera hybrida*, we believe that this dimer is the same as theirs, i.e., a C_4_–C_4_ symmetrical dimer of oxidized catechin, which they identified by NMR.

#### 2.2.2. (+)-Gallocatechin

**Figure 6:**
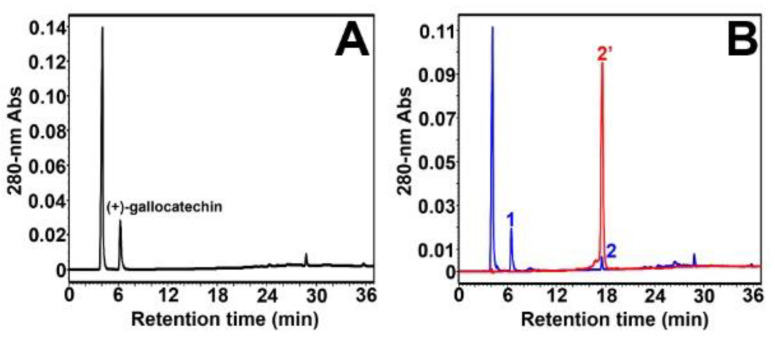
Reverse-phase HPLC analysis of *Vv*ANS product of (+)-gallocatechin. (**A**) HPLC analysis of the reaction mixture containing 100 µM (+)-gallocatechin without enzyme. (+)-Gallocatechin is eluted at 6.2 min, and no product is observed; (**B**) HPLC overlay chromatogram of the enzymatic products of (+)-catechin (in red) and the commercial standard of 100 µM cyanidin chloride (in blue). The blue peak 1 corresponds to (+)-gallocatechin (residual). The two large peaks observed at 4.0 min in (**A**) and (**B**) correspond to ascorbate.

(+)-Gallocatechin, which possesses a gallol group on ring B and therefore one more phenolic hydroxyl group than catechin (Figure 4), was tested next. A control experiment was also performed in the absence of *Vv*ANS (Figure 6A). A single enzymatic product (peak 2, 17.2 min) was observed upon incubation with *Vv*ANS (Figure 6B, in blue) with no residual substrate, indicating that (+)-gallocatechin is a substrate of *Vv*ANS that is more efficiently transformed by the enzyme than (+)-catechin. As shown in Figure 6B (in red), the single enzymatic product of (+)-gallocatechin could be assigned to delphinidin based on the HPLC retention time of the commercial standard of delphinidin chloride (peak 2′, 17.6 min); this was further confirmed by MS and MS/MS analyses using commercial delphinidin chloride as an external standard (Figure S4).

#### 2.2.3. (+)-Afzelechin and (+)-Catechin Stereoisomers

(+)-Afzelechin, which bears a single phenolic hydroxyl group on ring B and therefore one fewer than (+)-catechin, was tested next. Reverse-phase HPLC analysis revealed that no enzymatic product could be visualized. Similarly, no enzymatic product could be visualized with three stereoisomers of (+)-catechin, including (-)-catechin, (+)-epicatechin, and (-)-epicatechin (Figure S5).

### 2.3. Transformation of Flavanones by VvANS

Finally, flavanones, which are characterized by the absence of a substituent at C_3_, could be envisaged as potential substrates of *Vv*ANS, since naringenin, a member of this family, has been shown to act as a substrate of *At*ANS [35,43]. Therefore, naringenin was also tested as substrate of *Vv*ANS, and two different commercial standards of naringenin were used, including a racemic mixture of naringenin [(±)-naringenin] and a pure natural stereoisomer of naringenin (2*S*-naringenin). No enzymatic product could be visualized in either case (Figure S6), indicating that, unlike *At*ANS, *Vv*ANS does not recognize naringenin as substrate.

In summary, only five of the twelve tested polyphenols are substrates of *Vv*ANS, including three polyphenols of the family of dihydroflavonols and two polyphenols of the family of flavan-3-ols. The main information concerning the enzymatic transformation of these five substrates by *Vv*ANS is summarized in Table 1. The other six polyphenols, including (+)-epiDHQ, (-)-epiDHQ, (+)-afzelechin, (-)-catechin, (+)-epicatechin, and (-)-epicatechin, are not accepted by *Vv*ANS as substrates.

### 2.4. Real-Time MS Monitoring of the Enzymatic Transformation of (+)-Catechin

As above described, an ascorbate–cyanidin adduct and a dimer of cyanidin (or oxidized catechin) were observed as *Vv*ANS products of (+)-catechin. To try to clarify their formation mechanism, the enzymatic transformation of (+)-catechin was monitored by means of real-time mass spectrometry. However, the continuous injection of a reaction mixture containing free iron salt had to be avoided. Therefore, a *Vv*ANS-iron(II)-oxoglutarate complex was prepared extemporaneously (Figure S7) and used for the enzymatic transformation of (+)-catechin in the absence of free iron(II) salts. We had first checked that this *Vv*ANS-iron(II)-oxoglutarate complex was fully active, as shown by its ability to produce the same enzymatic product as the enzyme incubated in the presence of free iron(II) salt (Figure S8).

**Figure 7:**
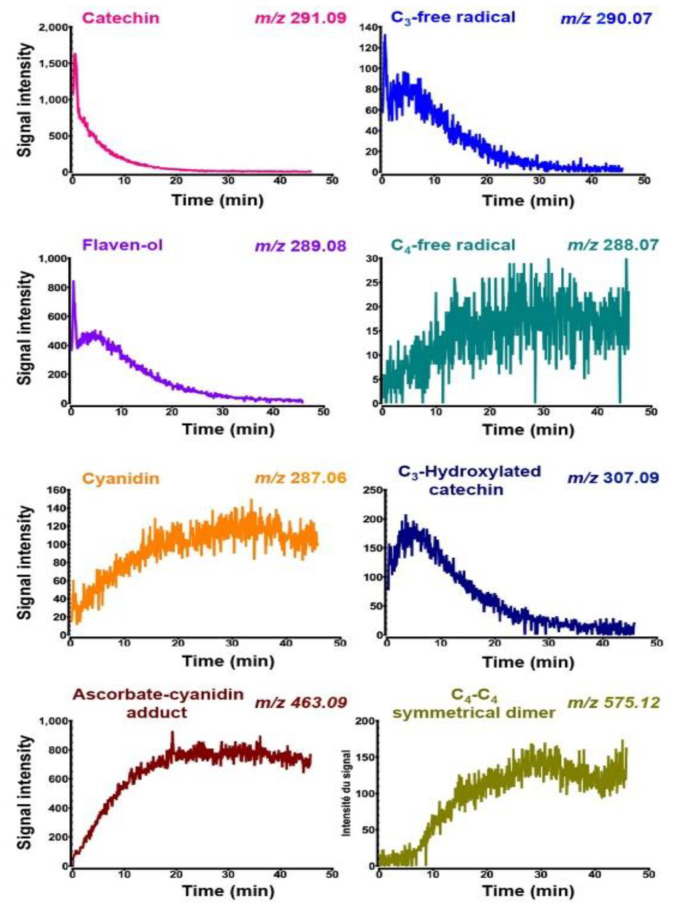
Time-course of (+)-catechin consumption and product formation by real-time mass spectrometry.

As shown in Figure 7, a rapid decrease of (+)-catechin is observed once the enzymatic reaction is initiated. Another molecule with *m*/*z* 290.07 decreases at about the same rate. It gives a much smaller signal, and it is not observed in the absence of enzyme, which means that it is most likely the one-electron oxidation product of catechin that is immediately formed by enzyme-catalyzed hydrogen abstraction, a generic step in iron/oxoglutarate oxygenases [44]. The next major event is the slower formation of four products (*m*/*z* 288.07, 307.09, 463.09, and 287.06). The first peak (*m*/*z* 288.07) has an extremely weak signal intensity and it corresponds to a three-electron oxidation product of catechin. The next peak (*m*/*z* 307.09) has a much higher signal intensity, and it is again clearly an intermediate rather than a stable end product. It can be assigned to a monohydroxylated product of (+)-catechin, based on its molecular mass (theoretical exact mass of the [M+H]^+^ ion: 290.08 + 17.00 = 307.08 Da), and it is most likely C_3_-hydroxylated (+)-catechin, i.e., a 3,3-gem-diol according to our previous study [32]. This intermediate is produced at a much higher initial rate than the other products, reaching a maximal concentration by approximately 5 min and then disappearing quite rapidly and becoming almost entirely consumed by 30 min, when the other products have reached their maximal values. This strongly suggests that such products are derived from this monohydroxylated intermediate.

The first two products immediately observed are the ascorbate–cyanidin adduct ([M+H]^+^, *m*/*z* 463.09) and cyanidin itself (M^+^, *m*/*z* 287.06), which is the four-electron oxidation product. A fourth product is the dimer of oxidized catechin ([M+H]^+^, *m*/*z* 575.12), but it is only observed after a lag time of at least 8 min. This suggests that the dimer is not directly produced from monohydroxylated catechin.

Based on signal intensities, it is likely that the ascorbate–cyanidin adduct is a major product in this continuous flow experiment, whereas cyanidin and the dimer of oxidized catechin are two minor products.

The two radical species (*m*/*z* 290.07 and 288.07) were not detected after 30 min in the reactional medium coupled to HPLC, which is expected for species with a rather short half-life. More importantly, the hydroxylation product of (+)-catechin had not been observed either in the reactional medium coupled with HPLC analysis (Figure 5B), which means that after a 30 min incubation period, the residual level of this intermediate was too low to be detected.

By contrast, the dimer of oxidized catechin, which is observed as a minor product in these experiments of continuous flow and real-time mass spectrometry, was a major product (together with the ascorbate–cyanidin adduct) in the reactional medium coupled with HPLC analysis (Figure 6B). There are at least two distinct experimental features that may be responsible for this. The first is that the reaction temperature was 35 °C in the reactional medium coupled with HPLC analysis, whereas it had to be fixed at 22 °C for continuous flow MS experiments. This suggests therefore that the formation of the dimer of oxidized catechin could be temperature-dependent. Perhaps a free radical precursor with a longer half-life at 22 °C would favor the dimerization path. The second difference relates to the availability of dissolved oxygen. In the experiments coupled with HPLC analysis, the reaction medium is in a vessel open to ambient air and under continuous stirring for 30 min, which means that the initial concentration of dissolved oxygen (230–260 µM under 1 Atm) is not limiting. By contrast, in the real-time MS experiment, the reaction medium is introduced at t_0_ from a tight syringe with no headspace, meaning that the initial concentration of dissolved O_2_ is limiting, which may result in a faster decrease in oxygen that might differently affect the yield of each product. For example, a free-radical mediated carbon–carbon dimerization would be more efficient at lower oxygen concentration where the formation of peroxyl radicals is less favorable. More generally, this oxygen-limiting experimental setup is probably the reason why low yet significant amounts of free radical intermediates (*m*/*z* 290.07 and *m*/*z* 288.07) are detected by MS.

## 3. Discussion

### 3.1. Structure–Activity Relationships

*Vv*ANS possesses a wide range of substrates, as expected from what had been observed with ANS from *Arabidopsis thaliana* and *Gerbera hybrida* [35,40,41,43], although some differences were observed with *Vv*ANS, such as the absence of transformation of naringenin, and the transformation of (+)-catechin into an ascorbate–cyanidin adduct. Based on our study, the following structure–activity relationships can be formulated for *Vv*ANS:

(1) Only flavan-3-ols of the (2*R*,3*S*) configuration with either two phenolic hydroxyl groups (catechol) or three (gallol) on ring B are recognized by *Vv*ANS as substrates. This is the case for (+)-catechin (catechol) and (+)-gallocatechin (gallol). The requirement of a catechol or gallol on ring B is confirmed by (+)-afzelechin, which is not a substrate of *Vv*ANS, despite the required (2*R*,3*S*) configuration. The requirement for this (2*R*,3*S*) configuration is confirmed by the absence of transformation of (-)-catechin, (+)-epicatechin, and (+)-epicatechin, despite their catechol on ring B.
(2) Only dihydroflavonols of the (2*R*,3*R*) configuration are accepted as substrates by *Vv*ANS. Contrary to flavan-3-ols, the presence of a catechol or gallol group is not mandatory for dihydroflavonols, as shown by (+)-DHK, which is a good substrate of *Vv*ANS, despite its single phenolic hydroxyl group on ring B. The requirement for a (2*R*,3*R*) configuration is confirmed with (+)-epiDHQ and (-)-epiDHQ, which do not have a configuration (2*R*,3*R*) and cannot be transformed by *Vv*ANS.
(3) Naringenin is not substrate of *Vv*ANS, most likely because of the absence of hydroxyl group at C_3_, although this is not what has been found with *At*ANS.

Flavonols only exist as glucosides in grape berries, and the most abundant flavonol glucosides observed in Cabernet sauvignon grape berries are derived from quercetin and myricetin [45]. *Vv*ANS does not more efficiently to produce flavonols that are di- or tri-hydroxylated on ring B, such as quercetin and myricetin, but it does produce them, which may have some physiological significance.

### 3.2. Lack of Reliable Kinetic Data

Although multiple kinetic experiments have been performed over the years in our laboratory with dihydroflavonol reductases, leucoanthocyanidin reductases, and anthocyanidin reductases, we were not able to measure steady-state initial rates with *Vv*ANS to estimate distinct k_cat_ and K_M_ values for flavan-3-ols and dihydroflavonols. There are several reasons for this. The first reason is that our products cannot be specifically monitored by spectrophotometry, except for cyanidin, which is not a major and single product. Moreover, the instability of cyanidin (half-life close to 30–40 min in our conditions) is incompatible with a steady-state initial rate measured over more than a few minutes, which would also be the case with delphinidin. An alternative is to monitor oxygen consumption, but we could not prepare stock solutions of active oxoglutarate–enzyme complex at concentrations higher than 2 µM. We had therefore to initiate the reaction by adding the polyphenolic substrate in an ethanolic or methanolic stock solution that invariably resulted in a transient increase in apparent dissolved oxygen, which perturbed the signal for several minutes with either an oxygen Clark electrode or an optical fiber-based oximeter (see Figure S9). We therefore could not measure initial rates at steady-state by oximetry. To the best of our knowledge, no steady-state kinetics have ever been reported with purified anthocyanidin synthases.

### 3.3. Anthocyanidin Production and Hypothetical Reactional Mechanism Involved in (+)-Catechin Transformation

**Figure 8:**
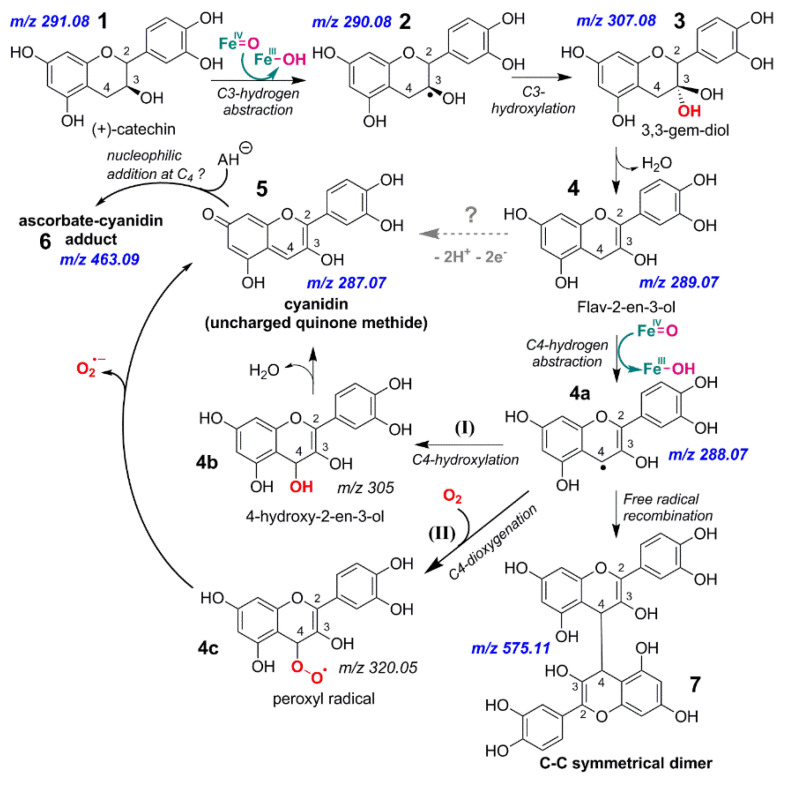
Hypothetical reactional scheme for *Vv*ANS transformation of (+)-catechin. 2-oxoglu = 2-oxoglutarate; AH_2_ = ascorbic acid; All *m*/*z* refer to (M+1) cationic species, and *m*/*z* which were observed by MS are in bold blue. The expected iron oxidation states of the enzyme active site are indicated only for hydrogen abstraction steps. Note that the flavylium cation of cyanidin would have the same *m*/*z* 287.06 as that observed for the mono-protonated form of the uncharged quinone methide since they are tautomers.

Overall, the real-time MS analysis, which was summarized in Figure 7, enables us to visualize an initial hydroxylated intermediate ([M+H]^+^, *m*/*z* 307.09) from which plausible sequences of product formation are summarized in Figure 8. An initial C_3_-hydrogen abstraction is catalyzed by *Vv*ANS on (+)-catechin 1 to produce the transient C_3_-free radical 2 from which hydroxylated catechin 3 (3,3-gem-diol) is the first metastable intermediate produced by the enzyme. The corresponding 3,3-gem-diol is expected to dehydrate into an enediol with a double bond at C_2_-C_3_ (compound 4) or at C_3_-C_4_. However, the subsequent formation of cyanidin 5 (drawn in uncharged form here) and of the symmetrical C-C dimer 7 could be achieved only with a double bond at C_2_-C_3_ (compound 4). Cyanidin should predominate in its two uncharged tautomeric forms (quinone methide) in the reaction mixture (pH 6.3). They should have the same reactivity (electrophilic C_4_) and therefore only one of them has been drawn on Figure 8. The formation of a symmetrical C-C dimer 7 can only be conceived from the recombination of C_4_ free radicals (compound 4a). The latter should be produced again by hydrogen abstraction, a major catalytic step in iron/oxoglutarate oxygenases, which is generally associated with the transition Fe^IV^=O to Fe^III^-OH at the active site [44].

We therefore conclude that structures 1, 2, 3, 4, 4a, and 7 are all mandatory, but there are several possibilities for the formation of both cyanidin 5 and the ascorbate-cyanidin adduct 6 from such structures. The direct transformation of 4 to 5 by *Vv*ANS cannot be ruled out, but it should be considered as highly speculative in the absence of realistic mechanistic rationale. We think that the transformation of structure 4a into cyanidin is a plausible alternative, and two possibilities can be envisaged. The first is that of step (I), a *Vv*ANS-catalyzed C_4_-hydroxylation, which despite a change in regiospecificity from C_3_ to C_4_, would be classical for an iron/oxoglutarate oxygenase. The second is step (II), which relies on dioxygen addition to the C_4_-centered free radical to produce a peroxyl radical intermediate. The addition of dioxygen to carbon-centered free radicals is indeed known to be very fast [46]. With such a peroxyl radical intermediate, a fast irreversible extrusion of superoxide O_2_**^.-^** would be expected, because at pH 6.3, the non-enzymatic dismutation of superoxide is also fast [47]. Presumably, the short half-life of this putative peroxyl radical would imply that the corresponding M+1 ion with *m*/*z* 320 would not be visualized by mass spectrometry.

As for the formation of the ascorbate–cyanidin adduct 6, we tentatively suggest in Figure 8 that it could be formed by nucleophilic addition of the ascorbate anion AH^−^ to the electrophilic C_4_ center of cyanidin 5, although a free radical mechanism involving compound 4a cannot be ruled out. Ascorbate is a good one-electron donor that behaves as an essential cofactor of *Vv*ANS. Its generic function in iron/oxoglutarate/ascorbate oxygenases is to reduce Fe(III) to Fe(II), thereby preventing oxidative inactivation of the active site during catalysis.

We do not yet know the structure of the ascorbate–cyanidin adduct, and it should be underlined that it is not observed in the absence of enzyme. Moreover, no ascorbate–delphinidin adduct was observed when (+)-gallocatechin was used as a substrate.

Among the five substrates that were identified in this work, (+)-dihydroflavonols were only transformed into the corresponding flavonols, and (+)-gallocatechin was converted into a single product, delphinidin.

The *Vv*ANS-catalyzed oxidative transformation of (+)-catechin is more complex, since three products were observed, including an ascorbate–cyanidin adduct ([M+H]^+^, *m*/*z* 463.09), a dimer of oxidized catechin ([M+H]^+^, *m*/*z* 575.12), and cyanidin. However, the data obtained from real-time MS monitoring of the enzymatic reaction imply that these three products are all derived from an initial common intermediate that is a monohydroxylated (+)-catechin, and most likely a 3,3-gem-diol ([M+H]^+^, *m*/*z* 307.09), based on our recent work [32].

We cannot explain why the ascorbate–cyanidin adduct and the dimer of oxidized catechin are observed in these in vitro experiments with our recombinant *Vv*ANS even though they were never described in biological extracts. This might result from in vitro conditions that do not exactly reproduce the in vivo conditions (microenvironment, enzyme/substrate ratio, cofactors), including the possibility that in vivo, ANS is part of a multi-enzyme channeling complex from which the formation of such cyanidin by-products is negligible.

However, the fact that *Vv*ANS can more efficiently produce pure delphinidin from gallocatechin than cyanidin from catechin may have some physiological significance, since malvidin and its glucoside, which are derived from delphinidin, are considerably more abundant in *Vitis vinifera* than cyanidin and its glucoside [48,49].

At a minimum, this work demonstrates that flavan-3-ols are substrates of *Vv*ANS, and that significant amounts of anthocyanidins can be produced in vitro by the enzyme from such substrates, which is not the case with leucoanthocyanidins [32]. The formation of significant amounts of cyanidin from (+)-catechin has also been confirmed with ANS from *Gerbera hybrida* [41] and from *Arabidopsis thaliana* [43]. In addition, with metabolically engineered bacteria, the optimal production of anthocyanins also required catechin [50,51,52,53].

For years, ANS and LDOX (leucoanthocyanidin dioxygenase) have been implicitly considered as two distinct names of a single enzyme. More recently, a thorough investigation of functional genomics and purified enzymes in *Medicago truncatula* led to the conclusion that two distinct enzymes were able to produce anthocyanins in two parallel pathways with distinct regulations [54]: one that the authors called *Mt*LDOX (access nb: XP_003601080.1) accepted (+)-catechin as a substrate to produce transient cyanidin immediately reduced by ANR to (+)-epicatechin, and the other that the authors called *Mt*ANS (access nb: XP_003611189.1) accepted leucocyanidin as a substrate to produce transient cyanidin immediately glycosylated to anthocyanins or reduced by ANR to (-)-epicatechin. In their interpretation, (-)-epicatechin produced from leucocyanidin and *Mt*ANS would serve as a precursor unit of polymeric proanthocyanidins whereas (-)-epicatechin produced from (+)-catechin would serve as an extension unit.

Based on sequence alignments with BLASTp (see Figure S10), our *Vv*ANS (access nb: NP_001268147.1) is clearly much closer to *Mt*ANS (78% identities) than to *Mt*LDOX (42% identities), but our data strongly support its ability to use (+)-catechin as precursor of cyanidin rather than leucocyanidin.

We do not know which role *Vv*ANS might play in the production of starter and extension units of proanthocyanidins in grapes, but our results support the idea that leucoanthocyanidin reductase (LAR) of *Vitis vinifera* could play a major role in the biosynthetic pathway of anthocyanidins by transforming leucoanthocyanidins into flavan-3-ols, which would then be the immediate precursors of anthocyanidins in vivo as ANS substrates. Just as for ANS/LDOX, there are two homologs of LAR enzymes (LAR1 and LAR2) in grape, but their structures and activities are almost identical [54]. They are however not only producing flavan-3-ols since they also convert the C_4_-thioethers 4β-(S-cysteinyl)-catechin and 4β-(S-cysteinyl)-epicatechin into (+)-catechin and (-)-epicatechin, respectively [55].

Data obtained on purified enzymes are very useful to know which transformations should be taken into consideration, especially for bioproduction optimization. However, given the enzymatic promiscuity of major actors, such as LAR, ANS/LDOX, and even ANR [56], unraveling the complexity of the biosynthetic pathway of anthocyanins in plants remains a considerable challenge.

## 4. Conclusion

In conclusion, among twelve tested polyphenols, only three dihydroflavonols and two flavan-3-ols were accepted as substrates of *Vv*ANS, with structure–activity relationships, mechanistic information, and one technical innovation that may be summarized as follows:

(1) Only 2*R*,3*S*-flavan-3-ols having either a catechol group (two adjacent phenolic hydroxyl groups) or a gallol group (three adjacent phenolic hydroxyl groups) as ring B are accepted as substrates. A simple phenol is not accepted.
(2) Only dihydroflavonols of (2*R*,3*R*) configuration are accepted as substrates.
(3) *Vv*ANS is able to produce anthocyanidins from flavan-3-ols such as (+)-gallocatechin or (+)-catechin, but not from leucocyanidin.
(4) When (+)-catechin is used as a substrate, cyanidin is not the major product observed; instead, the main products include an ascorbate covalent adduct of cyanidin and a dimer. For this complex multiple transformation, an experimental setup has been developed to monitor substrate, intermediates, and products by real-time mass spectrometry. The results imply that all products are derived from an initial 3,3-gem-diol intermediate that undergoes 2,3-dehydration to a flav-2-en-3-ol. Further transformation of this structure into C-C-dimer, ascorbate adduct, or cyanidin most likely involves a C_4_-free radical intermediate whose fate may artificially favor the dimer and the adduct because of the time-dependent limitation on dissolved oxygen. Further investigations will be required to understand why such a reactional complexity is not observed with (+)-gallocatechin, but it is noteworthy that its single product, namely delphinidin, is the precursor of malvidin and its glucoside, which are considerably more abundant in grapes of *Vitis vinifera* than cyanidin and its glucoside.
(5) This work also shows for the first time that an active iron/oxoglutarate dioxygenase can be extemporaneously prepared by gel filtration in the form of an iron/oxoglutarate complex, which can then be used without introduction of free iron salt in the reactional medium.

## 5. Materials and Methods

### 5.1. Chemicals

(+)-Sodium L-ascorbate (≥98%), α-ketoglutaric acid sodium salt (≥98%), iron(II) sulfate heptahydrate (≥99%), catalase from bovine liver (powder), (±)-naringenin (≥95%), (+)-taxifolin (2*R*,3*R*-dihydroquercetin or (+)-DHQ, ≥85%), kaempferol (≥95%), quercetin (≥95%), myricetin (≥96%), (−)-catechin (2*S*,3*R*-catechin, ≥98%), and methanol (HPLC grade) were all purchased from Sigma-Aldrich. 2S-Naringenin (≥98%), (+)-afzelechin (2*R*,3*S*-afzelechin, ≥98%) and (+)-gallocatechin (2*R*,3*S*-gallocatechin, ≥98%) were purchased from ChemFaces (Wuhan, China). (+)-Catechin (2*R*,3*S*-catechin, ≥99%), (−)-epicatechin (2*R*,3*R*-epicatechin, ≥99%), cyanidin chloride (≥96%) and delphinidin chloride (≥97%) were purchased from Extrasynthèse (Genay, France). (+)-Dihydrokaempferol (2*R*,3*R*-dihydrokaempferol or (+)-DHK, ≥95%) was purchased from ArboNova (Turku, Finland). (+)-Dihydromyricetin (2*R*,3*R*-dihydromyricetin or (+)-DHM) was purchased from Sequoia Research Products Ltd. (Pangbourne, Berkshire, UK).

### 5.2. Enzyme Source and Design of Reaction Medium

Recombinant anthocyanidin synthase from Vitis vinifera (*Vv*ANS, untagged enzyme) was produced and stored as previously described [32].

Enzyme reactions were carried out with the holoenzyme (iron-free *Vv*ANS) in a reaction medium containing free FeSO_4_ as a source of iron(II) for the enzyme active site. Twelve polyphenols from three distinct families (dihydroflavonols, flavan-3-ols, and flavanones) were tested as substrates of *Vv*ANS: (+)-DHK, (+)-DHQ, (+)-epiDHQ, (-)-epiDHQ, (+)-DHM, (+)-afzelechin, (+)-catechin, (-)-catechin, (+)-epicatechin, (-)-epicatechin, (+)-gallocatechin, and naringenin. Among them, (+)-epiDHQ, (-)-epiDHQ, and (+)-epicatechin were not commercially available and were therefore produced in the laboratory. The first two stereoisomers were respectively synthesized from the acidic epimerization of (+)-DHQ and the enzymatic transformation of 3,4-trans-leucocyanidin [32]; the latter was produced from the enzymatic reduction of cyanidin by anthocyanidin reductase from *Vitis vinifera* (*Vv*ANR) [57]. Methanolic stock solutions (2 mM) of each of these polyphenols were freshly prepared and stored at 4 °C during experiments, and products were analyzed by HPLC and mass spectrometry (MS). The reaction mixture (2 mL, final volume) contained 20 mM ammonium acetate, 20 mM NaCl, 2 mM ascorbate, 1 mM 2-oxoglutarate (2OG), 10 µM FeSO_4_, 0.1 mg/mL catalase, and 10^−6^ M *Vv*ANS (≈ 80.6 µg), pH 6.3 at 35 °C. After pre-incubation of the reaction mixture at 35 °C for 5 min, the reaction was triggered by the addition of 100 µM polyphenol, and then incubated at 35°C for 30 min under gentle magnetic stirring. Our selection of 35 °C as working temperature was the best compromise that we found to avoid reaction times longer than 30 min while keeping undetectable thermal denaturation over 30 min within the 30–40 °C range when catechin was used as substrate [39]. Our final methanol concentration was always 5% (*v*/*v*), which did not alter enzyme activity since upon increasing this value to 10%, product yields were not modified when catechin was used as a substrate.

### 5.3. Reverse-Phase HPLC Analysis

After 30 min of reaction, a 100 µL portion of the reaction mixture was analyzed by reverse-phase HPLC using an Atlantis C18 column (5 µm, 4.6 × 250 mm; Waters, Milford, MA, USA) with the same HPLC parameters as previously described [32].

### 5.4. Tandem Mass Spectrometry (MS/MS) Analysis

HPLC elution fractions of the products were collected and analyzed by positive-ion electrospray ionization tandem mass spectrometry (ESI-MS/MS), using a Q-Tof Premier mass spectrometer (Waters, Milford, MA, USA) under conditions described by Zhang et al. [32]. We systematically used MS and MS/MS analysis of HPLC-purified products, as well as freshly prepared methanolic solutions of 2 mM commercial standards including kaempferol, quercetin, myricetin, cyanidin and delphinidin, in agreement with classical MS fragmentation analysis of polyphenols [58,59].

### 5.5. Real-Time MS Monitoring of the Enzymatic Transformation of (+)-Catechin

To avoid continuous-flow injection of iron(II) into the ESI source of the mass spectrometer, the transformation of (+)-catechin was carried out in a reaction mixture containing no free iron(II) salt using the extemporaneously prepared holoenzyme loaded with iron(II), further referred to as *Vv*ANS-Fe(II)-oxoglutarate complex.

The reaction mixture (2.5 mL, final volume) contained 20 mM ammonium acetate, 20 mM NaCl, 2 mM ascorbate, 1 mM 2OG and ≈ 10^−6^ M *Vv*ANS-iron(II)-oxoglutarate complex, pH 6.3 (at 22 °C), and the reaction was initiated by the addition of 100 µM (+)-catechin. Upon vortex homogenization within a few seconds, 300 µL of the reaction mixture was taken with a 500 µL Hamilton syringe and infused into the ESI source under a flow rate of 5 µL/min by means of a syringe pump. The reaction was then monitored by real-time MS in positive ion mode in conditions previously described [60].

## Figures and Tables

**Table 1:** Enzymatic products obtained from the reactions catalyzed by *Vv*ANS.

Polyphenolic Substrates				Observed Products		
Name		*m*/*z*	RT ^1^	Name	*m*/*z*	RT ^1^
Dihydroflavonols (2*R*,3*R*)	(+)-DHK	289.05	16.3	Kaempferol	287.05	22.3
	(+)-DHQ	305.05	13.9	Quercetin	303.05	20.1
	(+)-DHM	321.05	11.2	Myricetin	379.05	18.4
Flavan-3-ols (2*R*,3*S*)	(+)-catechin	291.06	9.0	Ascorbate–cyanidin adduct	463.07	5.5
				Cyanidin	575.11	10.5
	(+)-gallocatechin	307.07	65.2	Delphinidin	287.05	18.6

^1^ RT, retention time in reverse-phase HPLC analysis, min.

## Data Availability

Data reported and analyzed in this paper have been archived in laboratory notebooks and computer files at CBMN, University of Bordeaux.

## Supplementary Materials

The following are available online. Figure S1: Identification of the contaminant of the commercial product of (+)-DHK; Figure S2: MS and MS/MS analysis of the product visualized as peak 3 in Figure 3B; Figure S3: MS and MS/MS analysis of the product visualized as peak 4 in Figure 5B; Figure S4: MS and MS/MS analysis of the product visualized as peak 2 in Figure 6B; Figure S5: Reverse-Phase HPLC analysis of the transformation of (+)-afzelechin (A), (−)-epicatechin (B), (+)-epicatechin (C) and (−)-epicatechin (D) by *Vv*ANS; Figure S6: Reverse-Phase HPLC analysis of the enzymatic transformation of naringenin; Figure S7: Protocol of production of the *Vv*ANS-iron(II)-oxoglutarate complex; Figure S8: HPLC determination of the activity of the *Vv*ANS-iron(II) complex; Figure S9: Oxygen signal perturbation upon injection of methanolic solution of (+)-catechin; Figure S10: Sequence alignments of *Vv*ANS with ANS and LDOX from *Medicago truncatula*.

## Abbreviations

ANR: anthocyanidin reductase; ANS, anthocyanidin synthase; CHI, chalcone isomerase; CHS, chalcone synthase; DFR, dihydroflavonol reductase; DHK, dihydrokaempferol; DHM, dihydromyricetin; DHQ, dihydroquercetin; ESI, electrospray ionization; LAR, leucoanthocyanidin reductase; LDOX, leucoanthocyanidin dioxygenase; 2OG, 2-oxoglutarate; UFGT, UDP-glucose: flavonoid 3-O-glucosyltransferase.
