# Supplementary material for: Oxidative Transformation of Dihydroflavonols and Flavan-3-ols by Anthocyanidin Synthase from Vitis vinifera
Source: Molecules. 2022 Feb 3;27(3):1047. doi: 10.3390/molecules27031047 (PMC8839691; doi:10.3390/molecules27031047)
Supplement: Supplementary file 1 [file molecules-27-01047-s001.zip › molecules-1560812-supplementary.pdf]

Supplementary Material

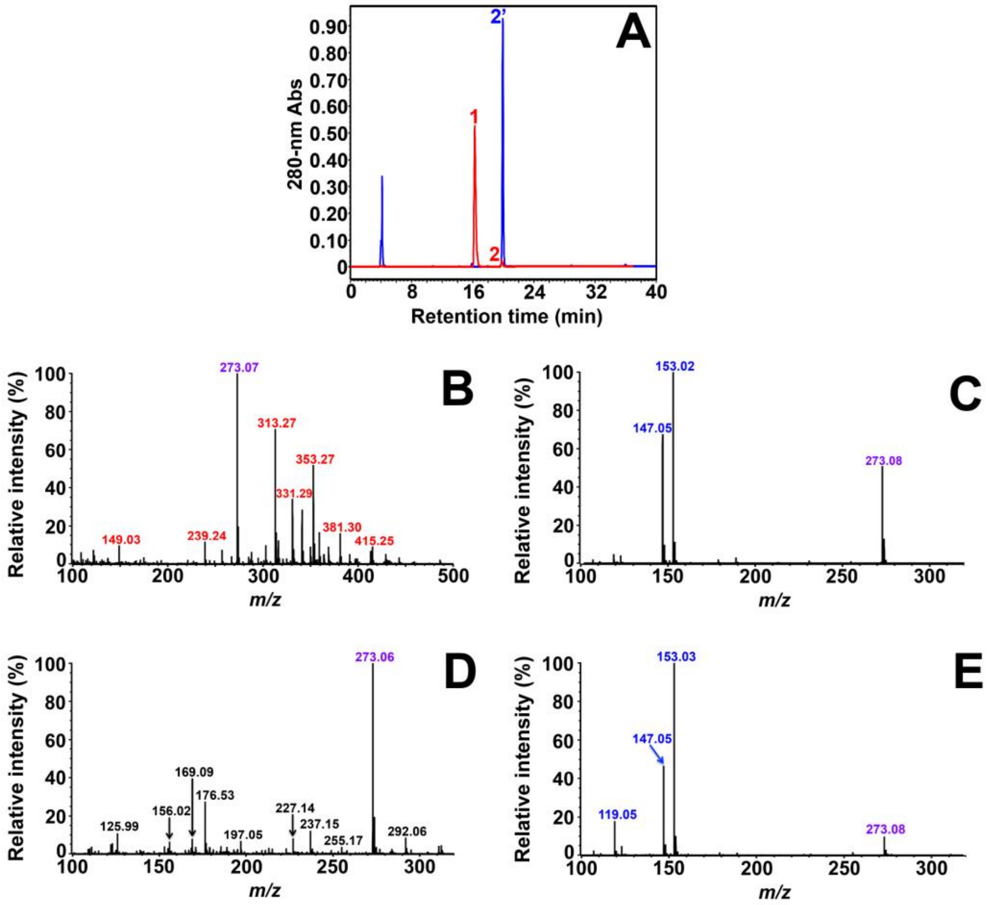

Figure S1. **Identification of the contaminant of the commercial product of (+)-DHK.**

The contaminant visualized as peak 2 was purified and collected during HPLC analysis, and then directly analyzed by MS and MS/MS. (**A**) HPLC analysis of the commercial standard of 100 µM (+)-DHK in the reaction mixture without enzyme (in red) and the commercial standard of 100 µM (±)-naringenin in methanol (in blue). The minor blue peak observed at 4 min corresponds to the ascorbate present in the reaction mixture as a cofactor of *Vv*ANS. (**B**) MS analysis of the collected peak 2. *m/z* 149, 239, 313, 331, 353, 381 and 415 are contaminant ions derived from phthalate or plasticizer in methanol extracts [42]. (**C**) MS/MS fragmentation of the $[M+H]^+$ ion with *m/z* 273 observed in (B). (**D**) and (**E**) are the MS and MS/MS spectra of commercial (±)-naringenin.

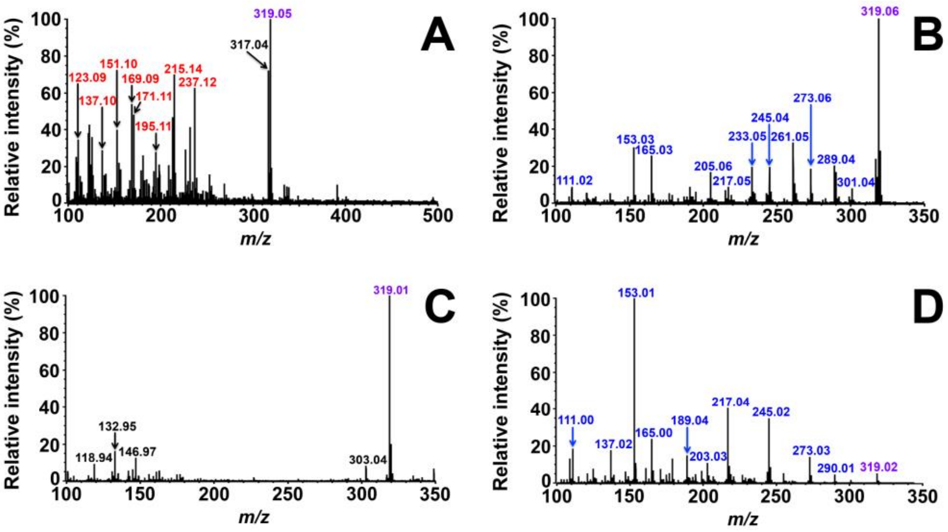

Figure S2. **MS and MS/MS analysis of the product visualized as peak 3 in Figure 3B.**

The product visualized as peak 2 was purified and collected during HPLC analysis, and then directly analyzed by MS and MS/MS. (**A**) MS analysis of the collected peak 3. *m/z* 123, 137, 151, 169, 171, 195, 215 and 237 are contaminant ions derived from phthalate or plasticizer in methanol extracts [42]. (**B**) MS/MS fragmentation of the $[M+H]^+$ ion with *m/z* 319 observed in (A). (**C**) and (**D**) are the MS and MS/MS spectra of the commercial standard of myricetin.

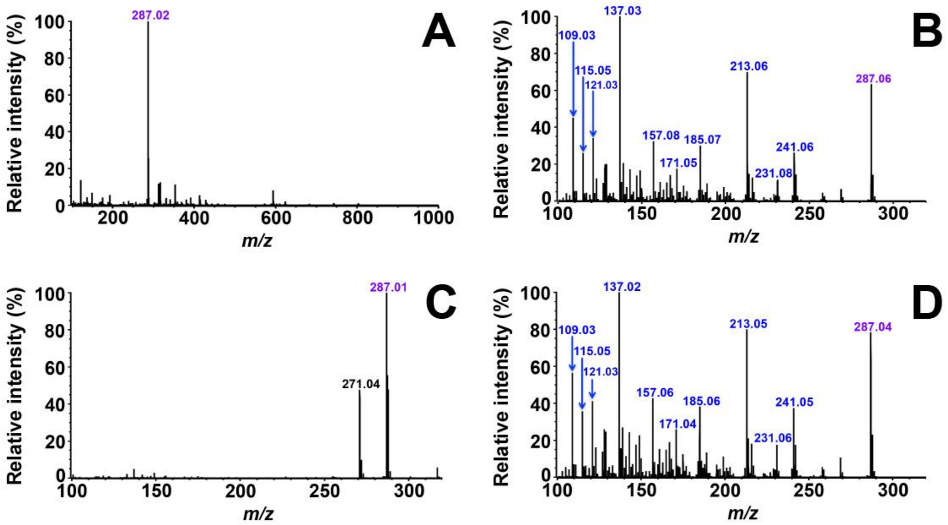

Figure S3. **MS and MS/MS analysis of the product visualized as peak 4 in Figure 5B.**

The product visualized as peak 4 was purified and collected during HPLC analysis, and then directly analyzed by MS and MS/MS. (**A**) MS analysis of the collected peak 4. (**B**) MS/MS fragmentation of the $[M+H]^+$ ion with *m/z* 287 observed in (A). (**C**) and (**D**) are the MS and MS/MS spectra of the commercial standard of cyanidin chloride.

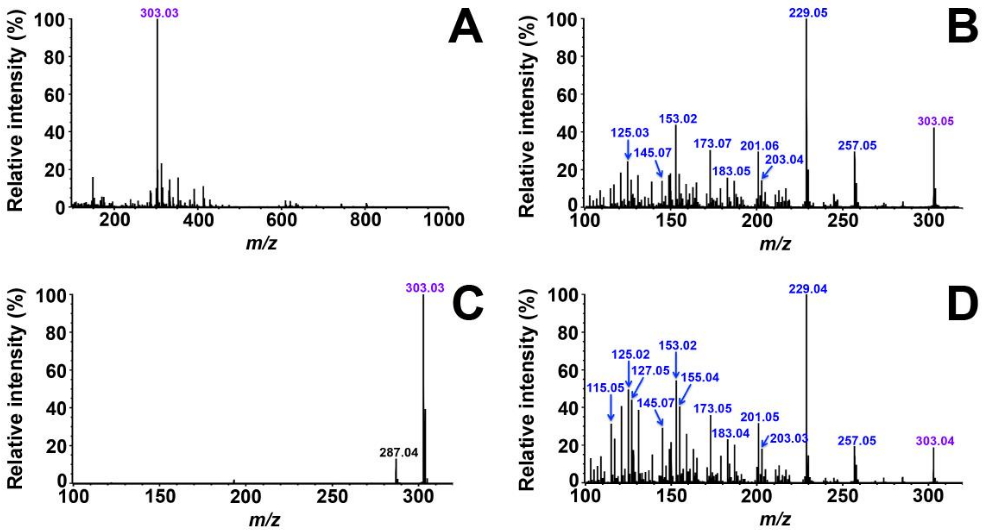

Figure S4. MS and MS/MS analysis of the product visualized as peak 2 in Figure 6B.

(**A**) and (**B**) are the MS and MS/MS spectra obtained from peak 2. (**C**) and (**D**) are the MS and MS/MS spectra of the commercial standard of delphinidin chloride.

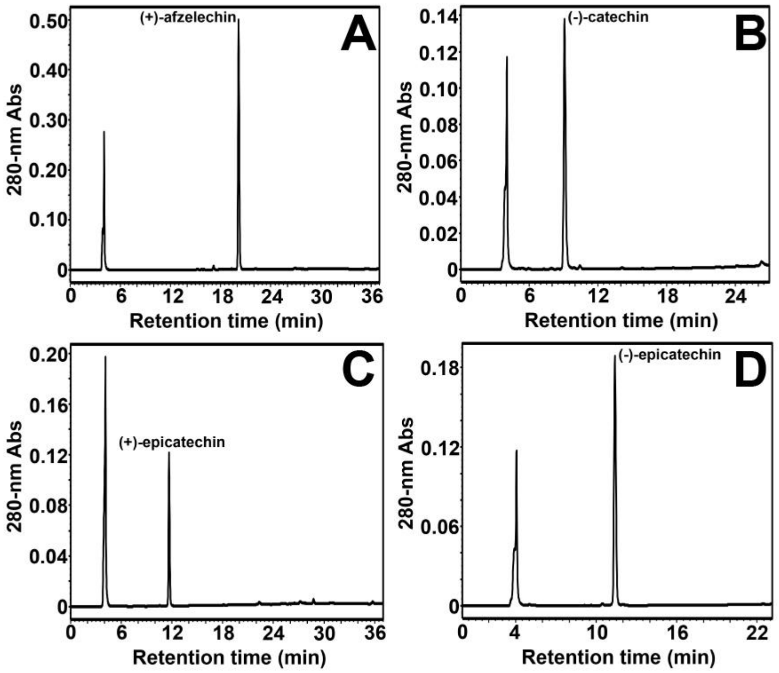

Figure S5. Reverse-Phase HPLC analysis of the transformation of (+)-afzelechin (A), (-)-epicatechin (B), (+)-epicatechin (C) and (-)-epicatechin (D) by *Vv*ANS.

The peak observed at 4 min in all cases corresponds to ascorbate present in the reaction mixture as a cofactor of *Vv*ANS, and no new product is observed after incubation with *Vv*ANS for each of these four flavan-3-ols, indicating that they are not substrates of *Vv*ANS.

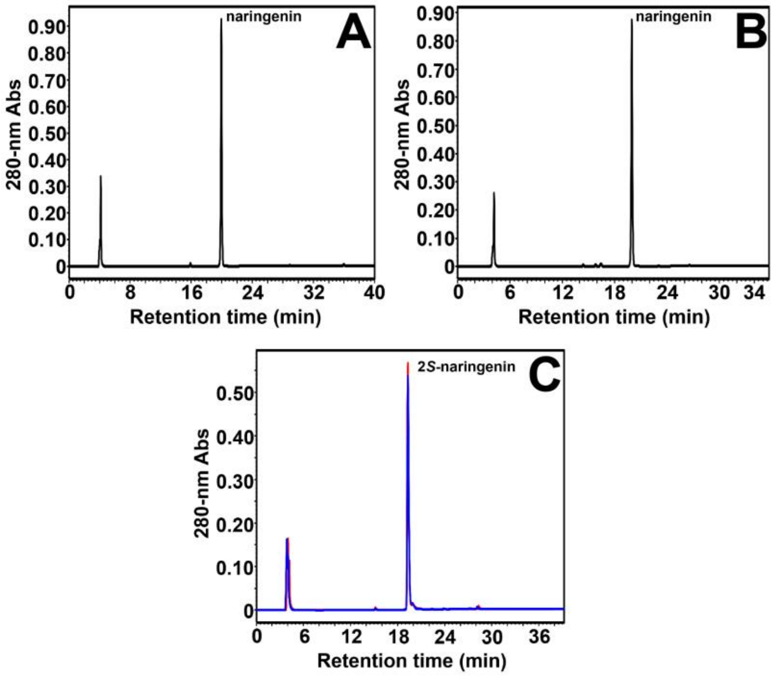

Figure S6. **Reverse-Phase HPLC analysis of the enzymatic transformation of naringenin.** **(A)** Commercial standard of 100 µM (±)-naringenin in the reaction mixture without enzyme. **(B)** Enzymatic transformation of 100 µM (±)-naringenin. (C) In red, commercial standard of 100 µM 2*S*-naringenin in the reaction mixture without enzyme; in blue, enzymatic transformation of 100 µM 2*S*-naringenin. The small peaks observed at about 4 min corresponds to ascorbate in all cases, and naringenin is eluted at about 20 min. No new products are observed upon the enzymatic degradation of both (±)-naringenin and 2*S*-naringenin, indicating that naringenin is not substrate of *Vv*ANS.

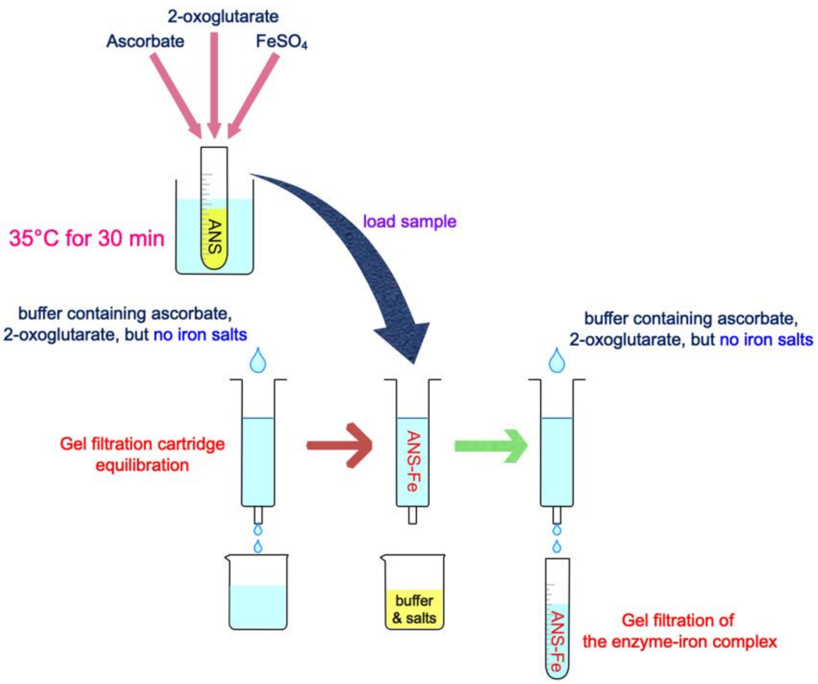

Figure S7. Protocol of production of the *Vv*ANS-iron(II)-oxoglutarate complex.

The production is carried out in 20 mM ammonium acetate containing 20 mM NaCl, and the pH is finally adjusted to 6.3 after the addition of 2-oxoglutarate (2OG) and ascorbate. The holoenzyme (*Vv*ANS, 10^{-6} M) is first incubated in the presence of 2OG (1 mM) which serves as a bidentate ligand of iron(II) at the active site of the enzyme at rest [44], and ascorbate (2 mM) which is a cofactor of ANS and which can also keep the iron salt (30 µM) in the form of iron(II) despite the presence of oxygen. The preformed *Vv*ANS-iron(II)-oxoglutarate complex is then loaded onto a gel filtration cartridge (PD-10 desalting column, GE Healthcare) and eluted with buffer containing 2OG (1 mM) and ascorbate (2 mM), but no iron salt. The gel filtration elution allowed us to collect the eluted *Vv*ANS-iron(II)-oxoglutarate complex before elution of the low molecular weight solutes, to avoid any free iron salt contamination.

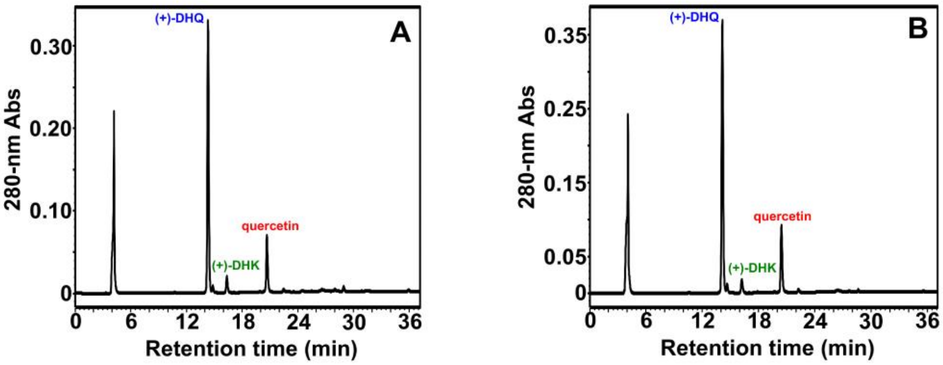

Figure S8. HPLC determination of the activity of the *Vv*ANS-iron(II) complex.

Quercetin was observed as the only enzymatic product of *Vv*ANS when (+)-DHQ was used as the polyphenolic substrate. Note that (+)-DHK was the contaminant of the commercial standard of (+)-DHQ. **(A)** Oxidative transformation of (+)-DHQ by the *Vv*ANS-iron(II) complex in the absence of iron(II) salt, and the enzymatic reaction was performed using the method described in section 3.2.1. **(B)** Oxidative transformation of (+)-DHQ by *Vv*ANS in the presence of iron(II) salt. The *Vv*ANS-iron(II)-oxoglutarate complex was produced using the protocol described in Fig. S7. The reaction mixture (2.5 mL, final volume) contained 20 mM ammonium acetate, 20 mM NaCl, 2 mM ascorbate, 1 mM 2OG and ≈ 10^{-6} M *Vv*ANS-iron(II) complex, pH 6.3 (at 35℃). The reaction was initiated by the addition of 100 μM (+)-DHQ, and then incubated at 35°C for 15 min under gentle magnetic stirring. A 100 μL portion of the reaction mixture was then analyzed by reverse-phase HPLC.

Figure S9. Oxygen signal perturbation upon injection of methanolic solution of (+)-catechin.

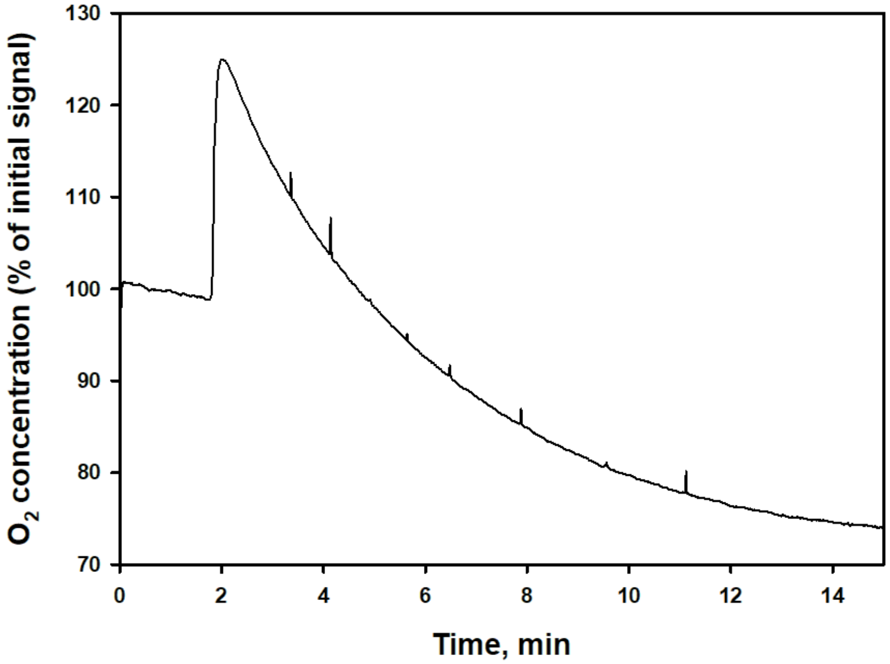

Monitoring of dissolved oxygen with an optical-fiber oximeter (FireSting-O_2, Pyroscience GmbH). A stock solution of 2 mM (+)-catechin was dissolved in 100% methanol. Upon addition of 125 µL of this stock solution into 2.250 mL of reactional medium containing 2OG, ascorbate and oxoglutarate-Fe^{II}-*Vv*ANS (≤1 µM) under magnetic stirring, a sharp jump is observed which prevents reliable measurement of true oxygen consumption for several minutes. In the absence of enzyme, a similar jump is observed, but it takes more than 4 min for the signal to return to a stable lower value. During these 4-5 min, if the enzyme is present, it consumes substantial but ill-defined amounts of O_2 and polyphenol. This prevents reliable measurements of initial rates at steady state. Similar results are obtained with ethanol instead of methanol, or with a Clark electrode instead of the FireSting-O_2.

Figure S10. Sequence alignments of VvANS with ANS and LDOX from Medicago truncatula

1. Query = Vv ANS ; Subject = Mt ANS (NCBI protein BLAST)

Score	Expect	Method	Identities	Positives	Gaps
569 bits(1466)	0.0	Compositional matrix adjust.	273/348(78%)	309/348(88%)	1/348(0%)

```
Query  2    VAVERVESLAKSGIISIPKEYIRPKEELESINDVFLEEKKEDGPQVPTIDLKNIESDDEK  61
               +RVESLA SGI SIPKEY+RPKEEL +I ++F EEKKE GPQVPTIDLK I S DE
Sbjct  3    TVAQRVESLALSGISSIPKEYVRPKEELANIGNIFDEEKKE-GPQVPTIDLKEINSSDEI  61

Query  62   IRENCIEELKKASLDWGVMHLINHGIPADLMERVKKAGEEFFSLSVEEKEKYANDQATGK  121
            +R  C E+LKKA+ +WGVMHL+NHGI  DL+ R+KKAGE FF L VEEKEKYANDQ++GK
Sbjct  62   VRGKCREKLKKAAEEWGVMHLVNHGISDDLINRLKKAGETFFELPVEEKEKYANDQSSGK  121

Query  122  IQGYGSKLANNASGQLEWEDYFFHLAYPEEKRDLSIWPKTPSDYIEATSEYAKCLRLLAT  181
            IQGYGSKLANNASGQLEWEDYFFH  +PE+KRDLSIWPKTP+DY + TSEYAK LR+LA+
Sbjct  122  IQGYGSKLANNASGQLEWEDYFFHCIFPEDKRDLSIWPKTPADYTKVTSEYAKELRVLAS  181

Query  182  KVFKALSVGLGLEPDRLEKEVGGLEELLLQMKINYYPKCPQPELALGVEAHTDVSALTFI  241
            K+ + LS+ LGLE  RLEKE GG+EELLLQMKINYYP CPQPELALGVEAHTDVS+LTF+
Sbjct  182  KIMEVLSLELGLEGGRLEKEAGGMEELLLQMKINYYPICPQPELALGVEAHTDVSSLTFL  241

Query  242  LHNMVPGLQLFYEGKWVTAKCVPDSIVMHIGDTLEILSNGKYKSILHRGLVNKEKVRISW  301
            LHNMVPGLQLFYEGKWVTAKCVPDSI+MHIGDT+EILSNGKYKSILHRGLVNKEKVRISW
Sbjct  242  LHNMVPGLQLFYEGKWVTAKCVPDSILMHIGDTIEILSNGKYKSILHRGLVNKEKVRISW  301

Query  302  AVFCEPPKDKIVLKPLPEMVSVESPAKFPPRTFAQHIEHKLFGKEQEE  349
            AVFCEPPK+KI+LKPLPE+V+ + PA+FPPRTFAQHI HKLF K++EE
Sbjct  302  AVFCEPPKEKIILKPLPELVTEKEPARFPPRTFAQHIHHKLFRKDEEE  349
```

2. Query = Vv ANS ; Subject = Mt LDOX (NCBI protein BLAST)

Score	Expect	Method	Identities	Positives	Gaps
271 bits(694)	5e-94	Compositional matrix adjust.	145/342(42%)	218/342(63%)	16/342(4%)

```
Query  4    VERVESLAKSGIISIPKEYIRPKEELESINDVFLEEKKEDGPQVPTIDLKNIESDDEKIR  63
            V+RV++LA + +  +P ++IR   E           K  +G  VP I L        +
Sbjct  3    VKRVQTLACNQLKELPPQFIRLANERPE------NTKAMEGVTVPMISL-------SQPH  49

Query  64   ENCIEELKKASLDWGVMHLINHGIPADLMERVKKAGEEFFSLSVEEKEKYANDQATGKIQ  123
               ++++ +A+ +WG   + +HGI   L++ ++  G+EFFSL  +EKE YAND ++GK
Sbjct  50   NLLVKKINEAASEWGFFVITDHGISQKLIQSLQDVGQEFFSLPQKEKETYANDPSSGKFD  109

Query  124  GYGSKLANNASGQLEWEDYFFHLAYPEEKRDLSIWPKTPSDYIEATSEYAKCLRLLATKV  183
            GYG+K+  N   ++EW DY+FHL  P  K +  +WPK+P  Y E   EY K +  +   +
Sbjct  110  GYGTKMTKNLEQKVEWVDYYFHLMSPHSKLNFEMWPKSPPSYREVVQEYNKEMLRVTDNI  169

Query  184  FKALSVGLGLEPDRLEKEVGGLEELLLQMKINYYPKCPQPELALGVEAHTDVSALTFILH  243
             + LS GL LE   L+  +GG EE+ L+MKIN YP CPQPELALGVE HTD+SA+T ++
Sbjct  170  LELLSEGLELESKTLKSCLGG-EEIELEMKINMYPPCPQPELALGVEPHTDMSAITLLVP  228

Query  244  NMVPGLQLFYEGKWVTAKCVPDSIVMHIGDTLEILSNGKYKSILHRGLVNKEKVRISWAV  303
            N VPGLQ++ +  WV    + +++ +HIGD LE+LSNG+YKS+LHR LVNKE+ R+SWAV
Sbjct  229  NDVPGLQVWKDNNWVAVNYLQNALFVHIGDQLEVLSNGRYKSVLHRSLVNKERKRMSWAV  288

Query  304  FCEPPKDKIVLKPLPEMVSVESPAKFPPRTFAQHIEHKLFGK  345
            F  PP + +V+ PLP +V+ ++PAKF  +T+A++  ++ F K
Sbjct  289  FVAPPHE-VVVGPLPPLVNDQNPAKFSTKTYAEY-RYRKFNK  328
```
